# Persistent Fibroadipogenic Progenitor Expansion Following Transient DUX4 Expression Provokes a Profibrotic State in a Mouse Model for FSHD

**DOI:** 10.3390/ijms23041983

**Published:** 2022-02-11

**Authors:** Darko Bosnakovski, David Oyler, Ana Mitanoska, Madison Douglas, Elizabeth T. Ener, Ahmed S. Shams, Michael Kyba

**Affiliations:** 1 Lillehei Heart Institute, 312 Church St. SE, Minneapolis, MN 55455, USA; oyler012@umn.edu (D.O.); mitan001@umn.edu (A.M.); dougl407@umn.edu (M.D.); elizabeth.ener15@gmail.com (E.T.E.); ashams@umn.edu (A.S.S.); 2 Department of Pediatrics, University of Minnesota, Minneapolis, MN 55455, USA; 3 Human Anatomy and Embryology Program, Faculty of Medicine, Suez Canal University, Ismailia 41111, Egypt

**Keywords:** facioscapulohumeral muscular dystrophy, DUX4, fibroadiopgenic progenitors, fibrosis

## Abstract

FSHD is caused by loss of silencing of the DUX4 gene, but the DUX4 protein has not yet been directly detected immunohistologically in affected muscle, raising the possibility that DUX4 expression may occur at time points prior to obtaining adult biopsies for analysis, with consequent perturbations of muscle being responsible for disease progression. To test the extent to which muscle can regenerate following DUX4-mediated degeneration, we employed an animal model with reversible DUX4 expression, the iDUX4pA;HSA mouse. We find that muscle histology does recover substantially after DUX4 expression is switched off, with the extent of recovery correlating inversely with the duration of prior DUX4 expression. However, despite fairly normal muscle histology, and recovery of most cytological parameters, the fibroadipogenic progenitor compartment, which is significantly elevated during bouts of fiber-specific DUX4 expression, does not return to basal levels, even many weeks after a single burst of DUX4 expression. We find that muscle that has recovered from a DUX4 burst acquires a propensity for severe fibrosis, which can be revealed by subsequent cardiotoxin injuries. These results suggest that a past history of DUX4 expression leads to maintained pro-fibrotic alterations in the cellular physiology of muscle, with potential implications for therapeutic approaches.

## 1. Introduction

Facioscapulohumeral muscular dystrophy is a common genetic disease of muscle affecting approximately 1 in 8300 individuals [1], and is caused by failure of silencing of the DUX4 gene. DUX4 is embedded within the D4Z4 macrosatellite repeat [2], which is present in a tandem array at the terminus of chromosome 4q, and most FSHD-causing mutations are integral contractions of the D4Z4 array [3,4], although second site mutations that affect the chromatin state of the array also contribute [5,6]. When these mutations impair the repeat-induced silenced chromatin state of D4Z4 [7,8], the potential for transcriptional “leakage” occurs, which if it arises on an allele providing a polymorphic poly-A signal [9] will lead to leaky expression of the DUX4 protein.

The cellular effects of DUX4 expression are bimodal: at high levels of expression, it causes apoptosis [10,11], which may be through various perturbations, including elevated oxidative stress [12] leading to DNA damage [13], expression of double stranded RNA [14], and/or abnormal hypoxia signaling [15]. At low expression levels, DUX4 impairs myogenic differentiation [12,16,17,18]. Despite several excellent monoclonal antibodies, DUX4 expression has not yet been demonstrated at the protein level in human FSHD muscle biopsy specimens. Whether this might be due to very low levels of expression, below the threshold for detection, or due to high levels of expression leading to immediate elimination of expressing cells, is currently debated. Neither model is very satisfying, as the expression of DUX4 is observed in rare myonuclei of cultured FSHD myoblasts and myotubes [19,20] and this rate of expression does not appear to correlate with the catastrophic loss of muscle tissue seen in certain muscles with FSHD.

The presence of the DUX4 protein in situ has been implicated indirectly by transcriptional profiling studies finding DUX4 target genes to be statistically elevated as a group in sets of FSHD patients compared to controls [21,22]. If the target genes tend to be elevated, it is difficult to imagine a model in which the protein is not playing a role. However, the correlation based solely on DUX4 target genes is not perfect, sometimes grouping controls with FSHD cases, and it requires multiple target genes and large group sizes to be detected. This stands in marked contrast to gene expression changes in muscle cells expressing DUX4 at detectable levels in vitro [12,23]. An alternative explanation for subtle DUX4 target gene differences in the absence of immunohistologically detectable DUX4 protein is that DUX4 protein was transiently expressed at some previous time, turned on a set of targets, and they did not completely return to baseline after DUX4 expression ended.

The possibility of past DUX4 expression leading to pathological changes that persist has implications for targeted therapy in FSHD. Current strategies are aimed at preventing current and future DUX4 expression, but if past expression has enduring consequences, therapies addressing the pathological state itself may be essential. To this end, we sought to test to what degree muscle returns to normal after windows of DUX4 expression, using the iDUX4pA mouse model. In this model, the DUX4 gene under the control of a Tet response elements (TRE) promoter can be induced selectively in muscle fibers, by way of the human skeletal actin promoter driving the rtTA transactivator, by doxycycline [24]. The reversibility of the Tet-On system makes this model uniquely suited to answering this question. We therefore designed a series of studies to test the extent of recovery after prior DUX4 expression, and to specifically evaluate any persistent pathological changes, and how they might impact the resilience of recovered muscle to later injury.

## 2. Results

### 2.1. Pulse DUX4 Induction Promotes a Long-Lasting Increase in the Frequency of Fibroadipogenic Progenitors

**Figure 1:**
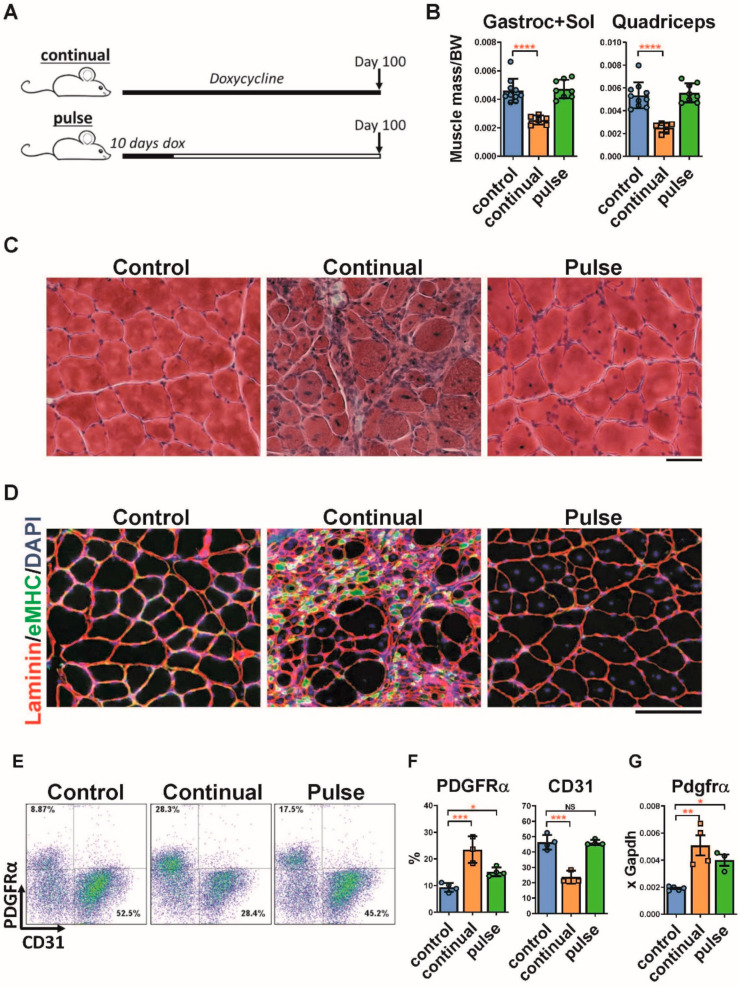
Elevated FAPs in the muscle after a pulse of DUX4. (**A**). Scheme of doxycycline induction protocol in iDUX4pA;HSA mice. Mice in one arm of the experiment continuously received doxycycline (chow containing 625 mg/kg doxycycline) for 100 days. This group is named ‘continual’. In the other arm, mice were fed with doxycycline chow for the first 10 days of the experiment and for the following 90 days received standard (doxycycline-free) chow. This group is referred to as ‘pulse’. (**B**). Mass of different muscles normalized to body weight from iDUX4pA;HSA mice continuously or pulse fed with doxycycline food. Data represent mean ± SEM, **** *p* < 0.0001 by one-way ANOVA, n = 6. (**C**). Representative H&E staining on quadriceps from control and iDUX4pA;HSA after continual or pulse induction with doxycycline. Scale bar 100 μm. (**D**). Immunofluorescence (IF) staining (bottom panel) for laminin (red), embryonic myosin heavy chain (eMHC, green) and nuclei (DAPI, blue) on the slides presented in (**C**). (**E**). Representative FACS plots for PDGFRα and CD31-expressing cells in the muscle of iDUX4pA;HSA mice pulse- or continuously treated with doxycycline. (**F**). Summary of the FACS analyses represented in (**E**). Data are expressed as mean ± SEM; * *p* < 0.05, *** *p* < 0.001, by one way ANOVA, n = 4. (**G**). RT-qPCR for *Pdgfra* in gastrocnemius from continuously and pulse induced mice. Data represent mean ± SEM; * *p* < 0.05, ** *p* < 0.01, by one-way ANOVA, n = 4.

In order to study the long-term recovery of muscle after transient DUX4 expression, we induced fiber-specific DUX4 expression by providing iDUX4pA mice doxycycline chow for 10 days, followed by switching back to normal chow (Figure 1A). Three months later (90 days after the 10 day pulse), muscle size had recovered back to normal (Figure 1B). However, histological examination revealed a mild extracellular matrix alteration accompanied by increased interstitial cell infiltration (Figure 1C). Evidence of the prior muscle damage was apparent in centrally located nuclei and more heterogeneous muscle fiber sizes (Figure 1C,D). In contrast, when mice were continually fed dox chow, severe muscle damage combined with ongoing evidence of muscle regeneration, seen by small embryonic myosin heavy chain positive myofibers, was obvious (Figure 1C,D). 

Previous long-term induction studies with the iDUX4pA mouse model showed that dystrophic muscle continually expressing DUX4 contained significantly elevated numbers of inflammatory cells as well as PDGFRα+ fibroadipogenic progenitors (FAPs), and reduced numbers of CD31+ endothelial cells and diminished microvasculature [25]. FACS analyses of muscle 3 months post-DUX4 pulse revealed that the frequency of inflammatory cells had declined to WT levels (Supplementary Figure S1), however the frequency of PDGFRα+ cells remained elevated (Figure 1E,F). Elevated expression of *Pdgfrα* in the muscle was also confirmed by RT-qPCR (Figure 1G). Mice continuously treated with doxycycline showed progressive muscle atrophy and FAP expansion. 

To determine the dynamics of these events, we performed a time course analysis over the first 20 days post-pulse (Supplementary Figure S2A). This revealed that Pdgfrα+ cells were most elevated immediately after the pulse when muscle size was most affected and the reduction of CD31+ cells the most severe (Supplementary Figure S2B). However, while the number of CD31+ cells progressively recovered to nearly normal over the 20 days, the number of FAPs remained steadily elevated (Supplementary Figure S2C,D). Remarkably, elevated FAPs persisted even to 5 months post pulse induction (Supplementary Figure S3).

### 2.2. Pulse Induction Leads to Fibrosis

**Figure 2:**
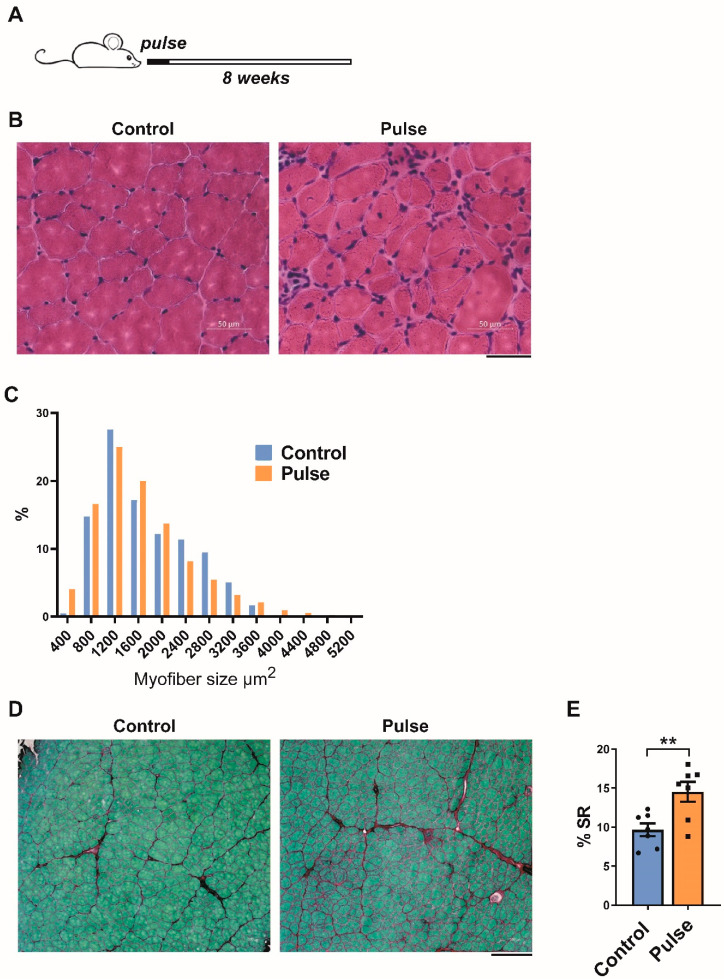
Fibrosis and myofiber size alterations 2 months after a 10 day pulse of DUX4. (**A**). Scheme of doxycycline pulse induction protocol in iDUX4pA;HSA mice. Mice received a pulse of doxycycline for 10 days. Muscles were analyzed histologically after 8 weeks of recovery, a time point at which they were 3 months of age. (**B**). Representative H&E image of TA muscle of iDUX4;HSA mice that were not doxycycline-induced (control) or pulse-induced mice (pulse). Scale bar 50 μm. (**C**). Myofiber size distribution in TA of control and pulse-induced mice. X-axis represents myofiber size distribution bins. (**D**). Representative images of Sirius Red/Fast Green staining of quadriceps from WT and iDUX4pA;HSA mice 8 weeks after a 10 day pulse of DUX4. Scale bar 100 μm. (**E**). Quantification of deposition of fibrous tissue in the muscle presented in (**C**). Data represent mean ± SEM; ** *p* < 0.01 by *t*-test, n = 6.

To quantify histological changes, iDUX4pA mice were pulsed with dox chow for 10 days, followed by a 2 month recovery period and compared to mice that were not pulse-induced (Figure 2A). DUX4 pulsed muscle appeared relatively healthy, with some centronucleation and expanded endomysium (Figure 2B). Fiber size in the tibialis anterior was notably smaller compared to the control (Figure 2C). To evaluate collagen content, we performed Sirius Red/Fast Green staining on quadriceps and quantified the cross-sectional area stained red (Figure 2D). This revealed a statistically significant increase in collagen content two months after a DUX4 pulse.

### 2.3. Muscle Recovery Is Diminished after Longer or Multiple Pulses

**Figure 3:**
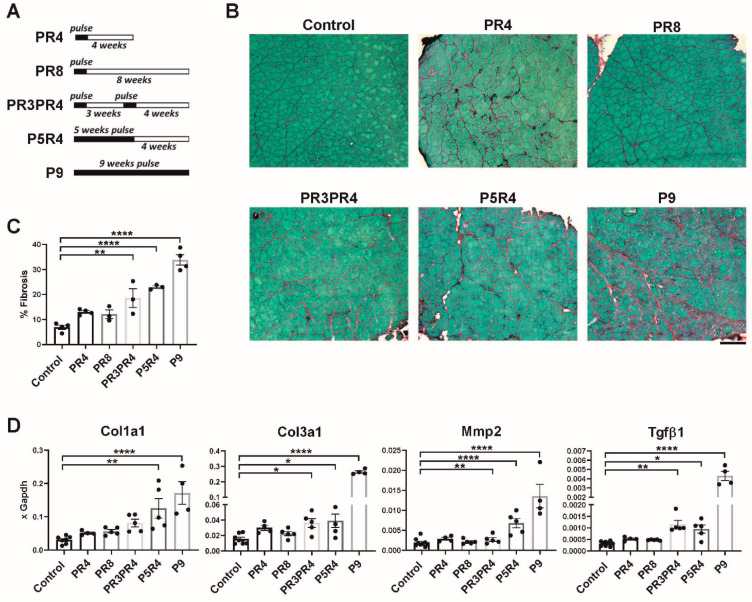
(**A**) Scheme of doxycycline pulse induction protocol in iDUX4pA;HSA mice. Mice received either a 10 day pulse, two such pulses, or a 5 week pulse. Muscles were analyzed histologically at the end of the indicated period. A continually induced control is indicated as a ‘9 weeks pulse’. (**B**) Representative Sirius Red/Fast Green images of TA muscle of iDUX4;HSA mice exposed to the different pulses of DUX4 shown in (**A**). Scale bar 100 μm. (**C**) Quantification of deposition of fibrous tissue in the muscle represented in (**C**). (**D**). RT-qPCR for genes related to fibrosis in gastrocnemius from mice exposed to the different pulses of DUX4 shown in (**A**). Data represent mean ± SEM; * *p* < 0.05 ** *p* < 0.01, **** *p* < 0.0001, by one-way ANOVA.

To relate the extent of retained fibrosis after recovery to the duration of the DUX4 pulse, we set up an experiment comparing different pulse-chase regimens (Figure 3A). When the length of the pulse was extended to 5 weeks, followed by a month of recovery, or when two separated 10-day pulses were given, followed by a month of recovery, the extent of fibrosis was markedly increased (Figure 3B,C). Consistent with this, expression of relevant collagens and matrix metalloproteases was maintained at an elevated level, five weeks after recovery from multiple, or a single longer pulse of DUX4 (Figure 3D). Expression of DUX4 and mouse DUX4 target genes *Myo1g* and *Wfdc3* were not detected in control (iDUX4;HSA uninduced mice) and in 8 weeks post pulsed mice suggesting that the observed phenotype originates from the transient DUX4 expression and not from low levels of ‘leaky’ transgene expression (Supplementary Figure S4). These data show that endurance of histological or cytological changes is linked to the degree of severity of the pulse of DUX4 expression.

### 2.4. Long-Term Effects of a Single Burst of DUX4 Expression

**Figure 4:**
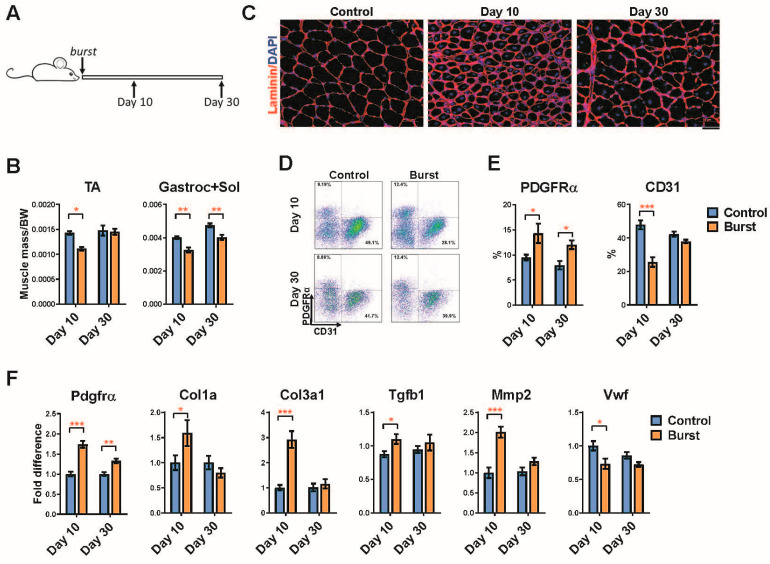
A burst of DUX4 is sufficient to induce long term FAP infiltration. (**A**) Scheme of doxycycline burst induction protocol in iDUX4pA;HSA mice. Mice at 4 weeks of age received a single dose of doxycycline (100 mg/kg, i.p.). Analyses were performed 10 and 30 days post-DUX4 induction. (**B**) Muscle mass normalized to the body weight from iDUX4pA;HSA mice 10 and 30 days after the burst induction. Data represent mean ± SEM, * *p* < 0.05, ** *p* < 0.01 by two-way ANOVA, n = 5. (**C**) Representative immunofluorescence images for laminin (red) and nuclei (DAPI, blue) in quadriceps at 10 or 30 days post-burst. Scale bar 50 μm. (**D**) Representative FACS profiles of FAPs (CD45^neg^/PDGFRα+) and endothelial cells (CD45^neg^/CD31+) in skeletal muscle at 10 and 30 days post-burst. (**E**) Summary of the FACS analyses on the samples represented in (**D**). Data represent mean ± SEM, * *p* < 0.05, *** *p* < 0.001 by two-way ANOVA, n = 4. (**F**). RT-qPCR for genes related to fibrosis and endothelial cells in gastrocnemius from iDUX4pA;HSA mice at the time of analyses. Data represent mean ± SEM, * *p* < 0.05, ** *p* < 0.01, *** *p* < 0.001 by two-way ANOVA, n = 5.

The studies above evaluated recovery from a 10 day period of transient DUX4 induction. To test the effects of the shortest possible period of expression, we treated animals with doxycycline via a single intraperitoneal (IP) injection (Figure 4A). We refer to this mode of induction as a ‘burst’ of DUX4. We observed a decline in muscle mass of both the TA and the gastrocnemius-soleus 10 days post-burst, which was fully recovered in the TA one month post-burst, and improved but was not fully returned at the 30 day time point in the gastroc-soleus (Figure 4B). The single burst causes muscle damage and regeneration as visualized by centrally nucleated small fibers, smaller fiber size and elevated extracellular matrix deposition (Figure 4C). We investigated the loss of endothelial cells and expansion of FAPs by flow cytometry and found the expected increase in frequency of PDGFRα+ cells and decrease in endothelial cells (CD31) 10 days post-burst (Figure 4D,E). By day 30, the number of endothelial cells had returned to normal, however the number of FAPs remained significantly elevated. The expression of genes associated with FAPs, endothelial cells, and fibrosis supported these findings (Figure 4F). This suggests that burst expression of DUX4 in fibers leads to long-term changes, well past the time point of DUX4 expression, particularly in the fibroadipogenic compartment, even when gross histology is not abnormal.

### 2.5. Burst Induction of DUX4 Sensitizes Muscle to Fibrosis following Later Injury

**Figure 5:**
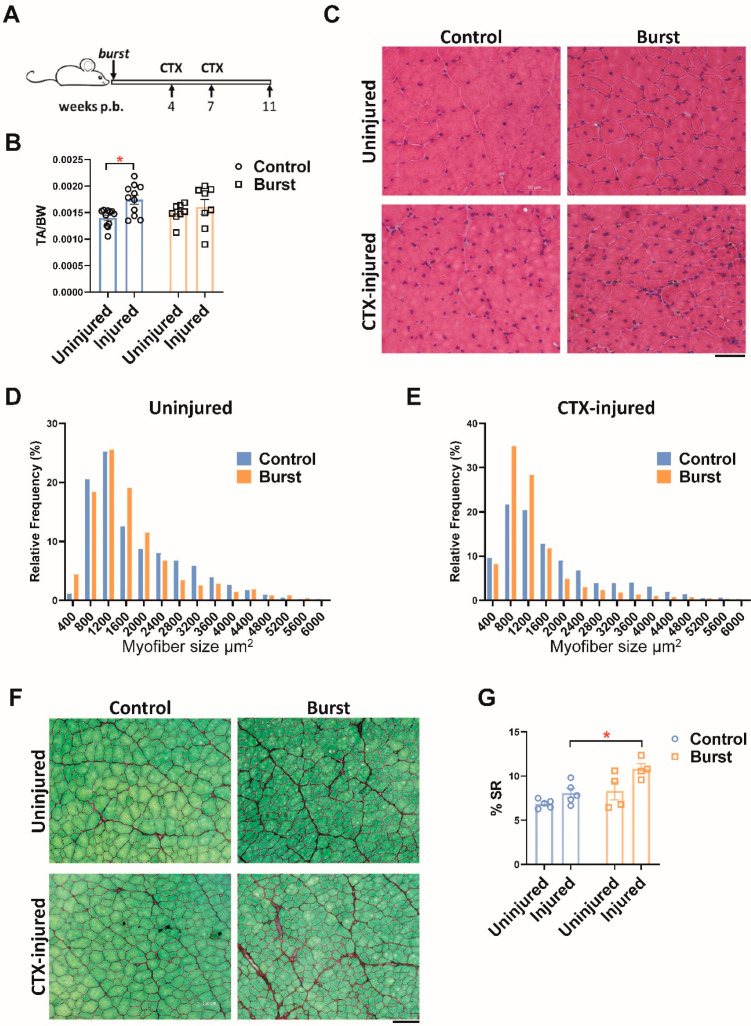
A burst of DUX4 sensitizes muscle to later injury. (**A**) Scheme of cardiotoxin (CTX) injury following DUX4 burst expression in iDUX4pA;HSA mice. Mice at 4 weeks of age received a single dose of doxycycline (100 mg/kg, i.p.) followed by two CTX injuries at 4 weeks post-burst and 7 weeks post-burst. Analyses were performed one month after the last injury. (**B**) TA muscle mass 11 weeks post-DUX4 burst. Data represent mean ± SEM, * *p* < 0.05, by two-way ANOVA, n = 8. (**C**) Representative image of H&E staining of TA muscle. Scale bar 50 μm. (**D**) Fiber size distribution at 11 weeks post-DUX4 burst. X-axis represents myofiber size distribution bins. (**E**) Fiber size at distribution 11 weeks post-DUX4 burst in CTX-injured muscles. X-axis represents myofiber size distribution bins. (**F**) Representative image of Sirius Red/Fast Green staining of TA muscle. Scale bar 100 μm. (**G**). Quantification of Sirius Red staining on the sections presented in (**F**). Data represent mean ± SEM, * *p* < 0.05, by two-way ANOVA, n = 4.

We wondered whether the elevated numbers of FAPs many weeks after a burst of DUX4 would have any effect on the way muscle responded to later injury. We therefore challenged TA muscles of mice that had been burst-induced with a single doxycycline injection by subjecting them to two rounds of cardiotoxin (CTX) injection, at 4 and 7 weeks after the burst. Muscle was analyzed one month after the final CTX injury (Figure 5A). This regimen of injury typically leads to hypertrophy of the TA muscle, and, indeed, in the control arm (not subjected to a DUX4 burst), we observed a significant increase in TA muscle mass. Notably however, this hypertrophy did not occur in mice previously subjected to the DUX4 burst (Figure 5B). Histologically, muscle that had been subjected to a prior DUX4 burst was more significantly disrupted by the CTX injuries than non-bursted muscle injured with CTX, with greater numbers of smaller fibers and a more prominent cellular infiltrate (Figure 5C–E). Fibrosis was quantified as previously, by Sirius Red/Fast Green staining, which revealed that a marked and significant increase in collagenous interstitial material had been provoked by the double CTX injury in the DUX4-burst group, but not in the control group (Figure 5F,G). Thus we conclude that a prior burst of DUX4 renders muscle prone to degenertion, which can be induced by external factors.

## 3. Discussion

The iDUX4pA;HSA mouse model is unique in that DUX4 expression is reversible, simply by withdrawing the inducer, doxycycline. An alternative inducible system, based on tamoxifen-sensitive cre, has been used to develop DUX4-expressing mice [26,27], however while DUX4 provokes muscle damage in those models, it does so continually and cannot be switched off. In order to determine whether and to what extent the phenotype provoked by DUX4 expression in myofibers is reversible, in the present study, we used transient expression strategies enabled by the iDUX4pA;HSA mouse, testing both pulse inductions of 10 days and burst inductions from a single injection of dox. In both cases, after the transient expression period, muscle recovered substantially. This result suggests the possibility that drugs targeting DUX4 expression or activity might be able to reverse some of the damage caused by DUX4 expression.

However, although histologically tremendously improved, muscle did not return to completely normal. Strikingly, both expression regimens led to an expansion of the FAP population, which persisted for as long as we were able to monitor. While the endothelial and inflammatory features returned to normal after dox withdrawal, the number of FAPs did not. This is not a typical response to injury, as while FAPs increase in the immediate aftermath of an acute injury, in the acute setting they return to normal quickly, for example within one week of a notexin injury [28]. Using the identical markers used in this study, we observed that the frequency of FAPs is unaltered 30 days post CTX-injury in WT mice [25]. Thus, the persistent expansion of the FAP population months after a single burst of DUX4 or a 10 day pulse of DUX4 suggests the possibility that homeostasis has been altered in a manner that favors fibrosis. Elevated numbers of FAPs are seen in various pro-fibrotic chronic muscle injury settings, including in *mdx* mice, as well as in mice subjected to denervation or chronic weekly BaCl_2_ injury [29,30]. The fibrotic propensity of muscle that had seen a previous burst of DUX4 was here revealed by later CTX injuries. This led to significant fibrosis, which is not normally seen after such CTX injury. The possibility that acquired muscle injury plays a role in the progression of FSHD is intriguing, as one of the features that distinguishes FSHD from most other muscular dystrophies is the frequent bilateral asymmetry in muscle deterioration. If DUX4 expression primed muscle for a dystrophic response to particular injuries, such that later injury initiated the fibrotic degeneration, then the stochastic nature of muscle injury might account for the asymmetry seen in FSHD.

Interestingly, in addition to an acquired propensity to fibrosis, the memory of a past DUX4 burst manifested also in failure of hypertrophy in response to CTX injury. The normal 10–15% increase in muscle mass provoked by the CTX injury was not observed in muscle that had previously experienced a DUX4 burst. Consistent with this result, both the burst and the 10 day pulse of DUX4 expression led to a shift in the myofiber size distribution towards smaller diameter fibers most obviously seen in the large increase in frequency of the very smallest fibers. An increased number of very small diameter fibers is a classic histological signature of FSHD-affected muscle [31].

It would be valuable to probe the various ways in which the perturbation of homeostasis due to past DUX4 expression might turn the muscle towards fibrotic degeneration. For example, different types of injury, including physiological injuries like forced eccentric contraction or excessive exercise, might push the muscle into fibrosis in the same way that CTX injury does. It would also be valuable to investigate approaches to inhibit the accumulation of FAPs and/or modulate their pro-fibrotic behavior.

The fact that transient expression of DUX4 has a very long-term, perhaps permanent, effect on muscle, has important ramifications for FSHD. The lack of immunohistological observation of DUX4 in situ in FSHD specimens to date makes a model involving transient past expression the more compelling. The possibility that a very early, perhaps even fetal, window of DUX4 expression may alter the homeostasis of muscle in such a way as to prime the tissue for later degeneration has significant implications. In terms of therapy, the current focus is on inhibiting DUX4 expression. While DUX4 is the obvious molecular target, the data presented here raise the possibility that the timing of inhibition may limit the effectiveness of such a therapy. Specifically, if expression early in life sets up a pro-fibrotic state that no longer needs DUX4 to drive disease progression, DUX4 inhibition would be unlikely to prevent continued muscle degeneration, and approaches to prevent the subsequent fibrotic processes would be favored. At the very least, the data suggest that strategies to address subsequent fibrotic processes should be considered alongside inhibition of DUX4 expression. The fact that DUX4 can barely be detected in patient biopsies, and indeed no publication has yet shown DUX4 protein on histological sections despite ongoing disease, is consistent with a model in which past DUX4 expression sets up a sustained pro-fibrotic and anti-regenerative disease state. The unique reversibility of the iDUX4pA;HSA mouse model will enable mechanistic investigations into this novel model for FSHD initiation and progression.

## 4. Methods

Mice: Four-week-old female mice carrying both the iDUX4pA and HSA-rtTA transgenes were fed doxycycline chow that contained 625 mg/kg doxycycline (ENVIGO). For DUX4 burst induction, a single dose (100 mg/kg) of doxycycline dissolved in PBS was injected intraperitoneally. In all experiments, iDUX4;HSA uninduced mice were used as controls.

Muscle histology: H&E and Sirius Red/Fast Green stainings were performed on 12 μm OCT-frozen muscle sections (TA and quadriceps) as previously described [24]. For immunofluorescence, tissue sections were fixed in 4% PFA for 10 min, permeabilized with 0.3% Triton X for 30 min and stained with primary antibody (anti-laminin (L9393, Sigma, St. Louis, MO, USA), anti-embryonic myosin heavy chain (eMHC, F1.652, Developmental Studies Hybridoma Bank, Iowa City, IA, USA), anti-DUX4 (MAB95351, R&D Systems, Minneapolis, MN, USA)) diluted in 3% BSA overnight at 4 °C. After 3 rounds of washing with PBS, the appropriate conjugated secondary antibody (Invitrogen, Waltham, MA, USA) was applied for 60 min at RT. Nuclei were visualized with DAPI (1:5000, Sigma). 

Quantification of fibrosis and myofiber sizing. The percent fibrosis in the TA was quantified on SR/FG-stained sections using ImageJ software. The image was first converted to grayscale based on green, and the threshold was adjusted. The area stained red was measured and normalized to cross-sectional area (CSA). The area of individual myofibers in the TA was quantified as described by [32]. Cellpose software was used to segment the images into individual myofibers. Fiber area was measured with the FIJI/ImageJ plugin, LabelsToROIs. 

FACS analyses: Muscles were digested with collagenase type II and dispase for 90 min. Mononuclear cells were stained with the following antibodies (all rat): PE-Cy7-CD45 (clone 30-F11, BD Biosciences, Franklin Lakes, NJ, USA), APC-CD31 (clone 390, eBioscience, San Diego, CA, USA), PE-Pdgfrα (CD140A, clone APA5, BD Biosciences), PE-Gr1 (clone RB6-8C5, eBioscience), APC-CD11b (M1/70, eBioscience), Pe-CD206 (clone C068C2, BioLegend, San Diego, CA, USA), PE-CD68 (clone FA-11, BioLegend) resuspended in PBS/1% FBS. Samples were run on a BD FACSAria instrument and data were analyzed using FlowJo (BD Biosciences). All experiments were done on at least 4 biological replicates.

RNA isolation, Quantitative Real Time RT-PCR (RTqPCR) and RNAseq: RNA was extracted using Trizol and RNA extraction kit (Zymo, Irvine, CA, USA) following the manufacture’s protocol. cDNA was made using 0.5 µg total RNA with oligo-dT primer and cDNA Synthesis Kit (Applied Biosystems, Waltham, MA, USA) following the manufacturer’s instructions. qPCR was performed by using Premix Ex Taq (Probe or SybrGreen qPCR, Takara, Kusatsu, Shiga, Japan) and commercially available probes from Applied Biosystems (*Gapdh*, Mm99999915_g1; *Vwf*: Mm00550376_m1; *Pdgfra*: Mm00440685_g1; *Myo1g*: Mm00617991_m1; *Wfdc3*: Mm01243777_m1) or custom-designed primers (*Col1a1*: F: 5′ GAG CGG AGA GTA CTG GAT CG and R: 5′ TAC TCG AAC GGG AAT CCA TC; *Col3a1* F: 5′ TGG TCC TCA GGG TGT AAA GG and R: 5′ GTC CAG CAT CAC CTT TTG GT; *Tgfb1* F: 5′ CTC CCG TGG CTT CTA GTG C and R: 5′ GCC TTA GTT TGG ACA GGA TCT G; *Mmp-2* F: 5′ CAA GTT CCC CGG CGA TGT C and R: 5′ TTC TGG TCA AGG TCA CCT GTC). Gene expression levels were normalized to that of Gapdh and analyzed with 7500 System Software using the ∆CT method (Applied Biosystems). 

Statistics: GraphPad Prism software was used to analyze the data. Differences between groups were evaluated by one-way or two-way analysis of variance (ANOVA) followed by Tukey’s post hoc tests. Differences were considered significant at *p*-values of 0.05 or lower.

## Data Availability

Not applicable.

## Supplementary Materials

The following supporting information can be downloaded at: https://www.mdpi.com/article/10.3390/ijms23041983/s1.
