# Supplementary material for: Persistent Fibroadipogenic Progenitor Expansion Following Transient DUX4 Expression Provokes a Profibrotic State in a Mouse Model for FSHD
Source: Int J Mol Sci. 2022 Feb 11;23(4):1983. doi: 10.3390/ijms23041983 (PMC8880758; doi:10.3390/ijms23041983)
Supplement: Supplementary file 1 [file ijms-23-01983-s001.zip › ijms-1537856-SI.pdf]

Supplementary Figure S1

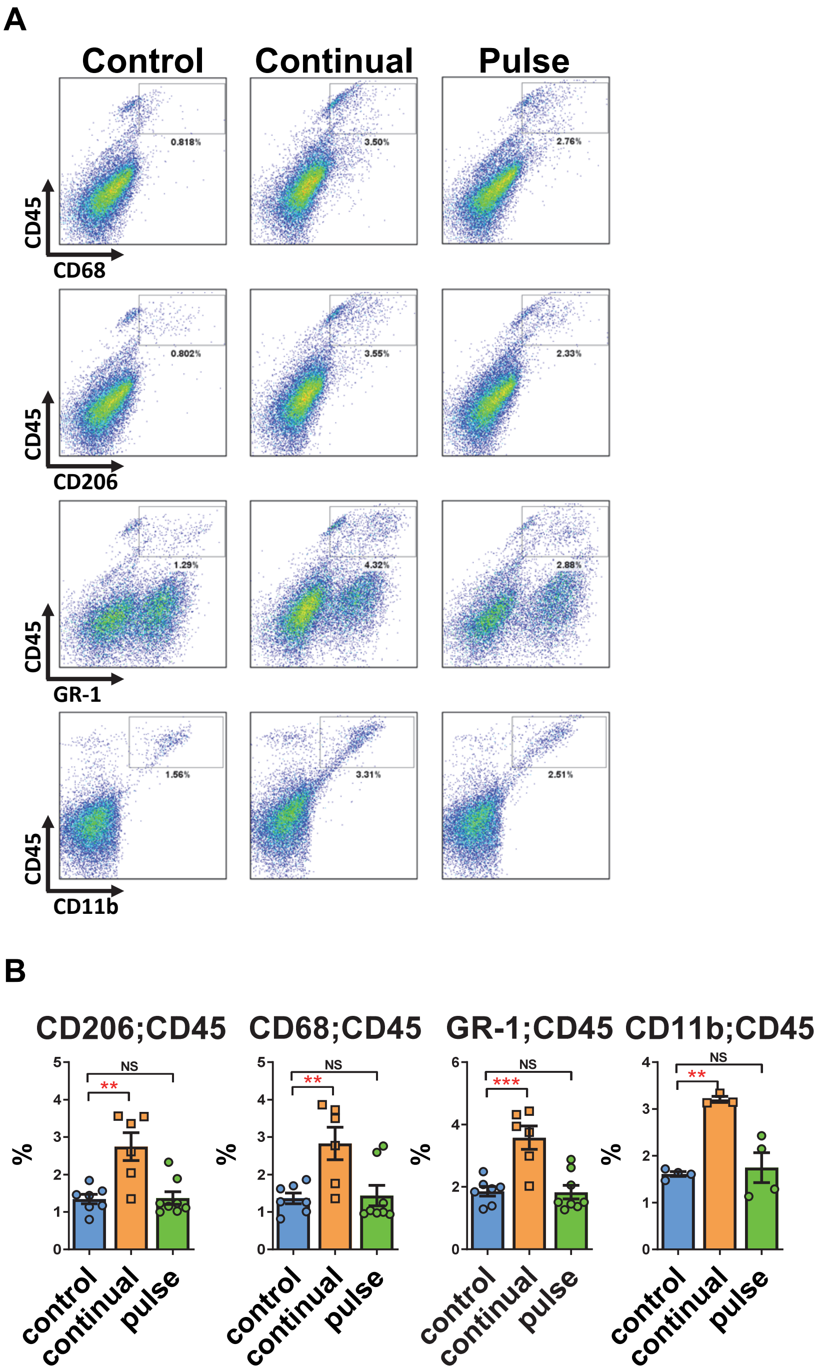

Supplementary Figure S1. Immune cell infiltration in the muscle in pulse induced mice

A. Representative FACS profiles of inflammatory cells (CD68, CD206, and Gr1) in the muscle of iDUX4pA;HSA mice pulse or continuously treated with doxycycline.

B. Summary of the FACS analyses represented in A. Data present mean ± SEM; **p<0.01, ***p<0.001, by one way ANOVA, n=5-9.

Supplementary Figure S2

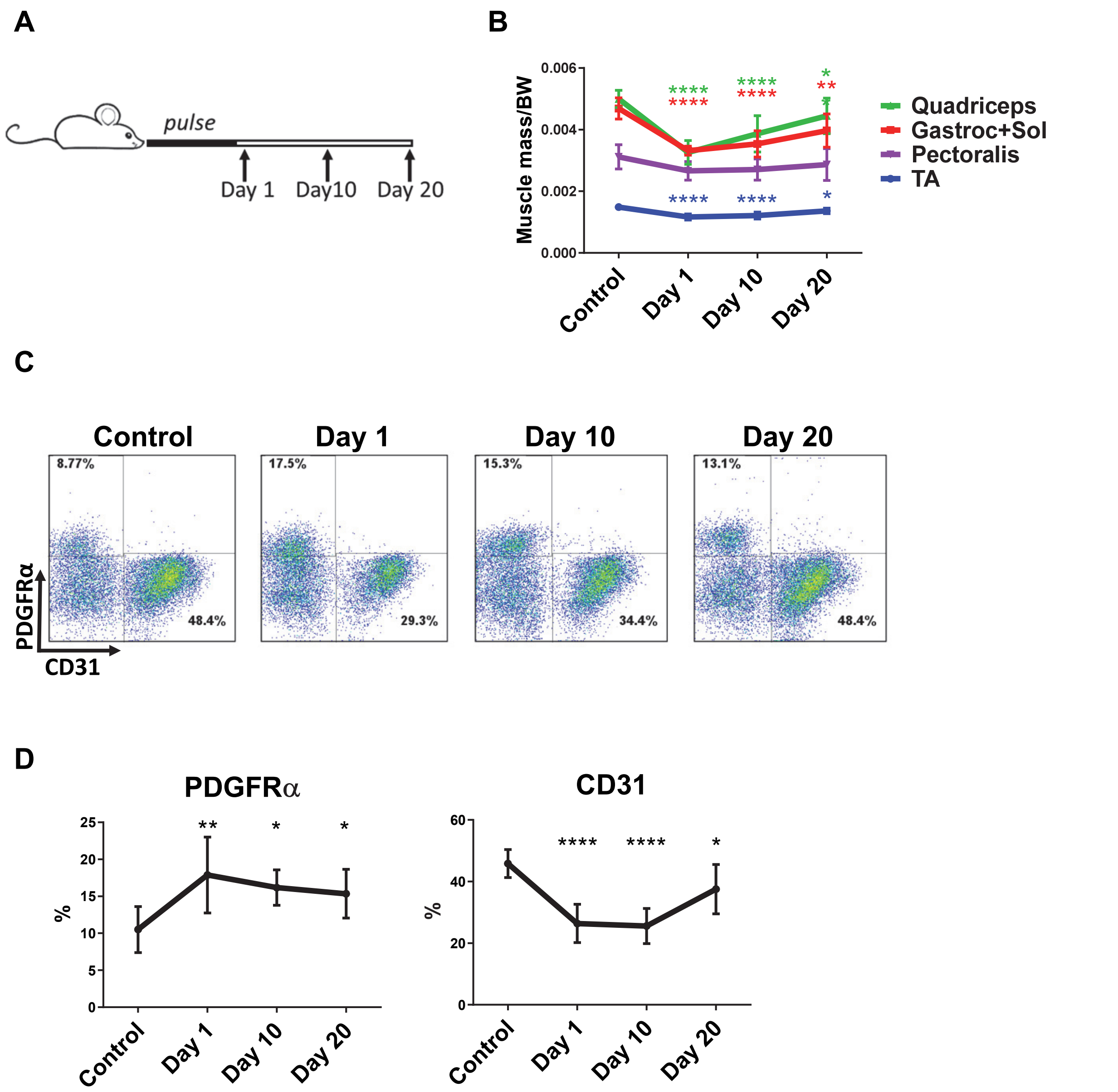

Supplementary Figure S2. Dynamics of FAPs infiltration after pulse of DUX4.

A. Scheme of doxycycline pulse induction protocol in iDUX4pA;HSA mice. Mice (4 weeks old) received doxycycline chow (625 mg/kg) for 10 days and the effect of induction was analyzed 1, 10 and 20 days post-induction.

B. Mass of different muscles normalized to the body weight from iDUX4pA;HSA mice pulse fed with doxycycline chow. Data represent mean ± SEM, *p<0.05, **p<0.01, ****p<0.0001 by one way ANOVA, n=6.

C. Representative FACS profiles of FAPs (CD45^{neg}/PDGFRα+) and endothelial cells (CD45^{neg}/ CD31+) in skeletal muscle at 1, 10 and 20 days post pulse induction.

D. Summary of the FACS analyses on the samples represented in C. Data present mean ± SEM, *p<0.05, **p<0.01, ****p<0.0001 by one way ANOVA, n=4.

Supplementary Figure S3

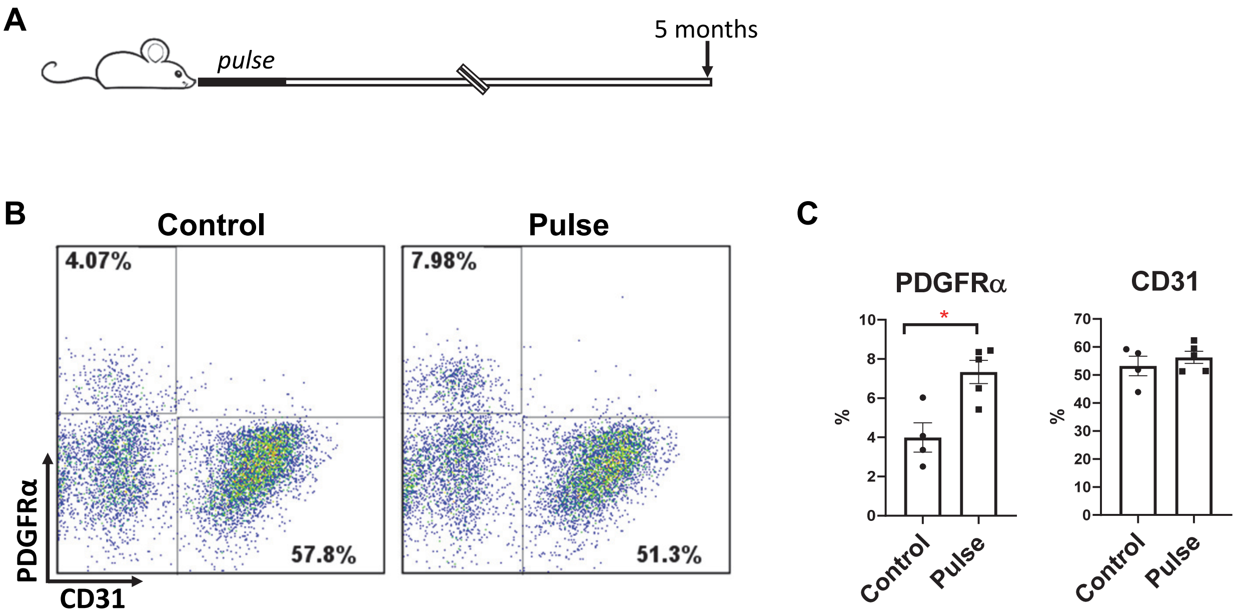

Supplementary Figure S3. Elevated FAPs in the muscle 5 months after a 10 day DUX4 pulse.

A. Scheme of pulse induction.

B. Representative FACS analyses for FAPs and endothelial cells in TA at 5 months after a 10 day pulse induction.

C. Summary of the FACS analyses in B. Data represent mean ± SEM; *$p<0.05$ by t-test, n=5.

Supplementary Figure S4

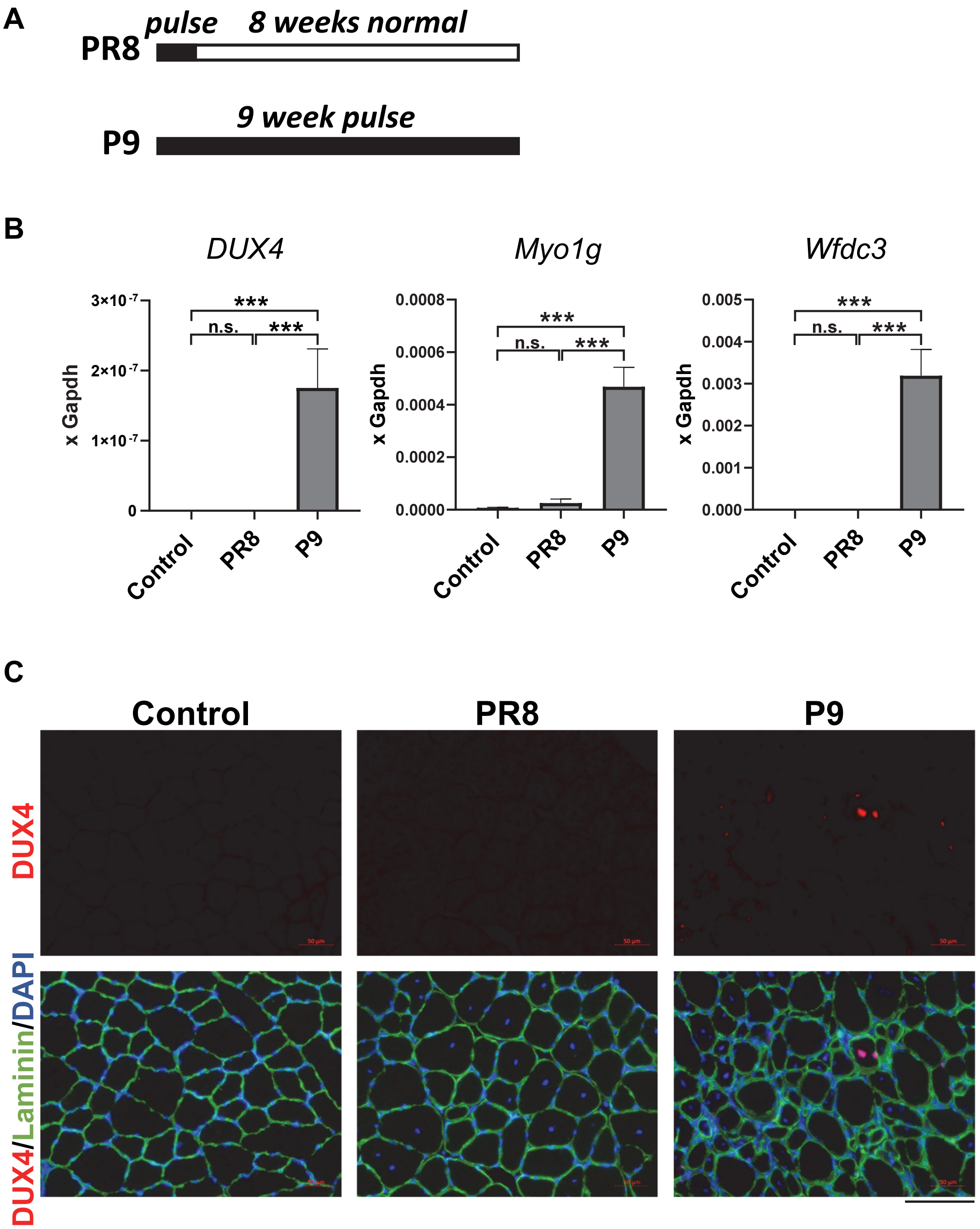

Supplementary Figure S4.

A. Scheme of doxycycline pulse induction protocol. Mice were transiently doxycycline induced for 10 days and analyzed 8 weeks later. A continually induced control is indicated as a '9 week pulse'.

B. RT-qPCR for DUX4 and DUX4 mouse target genes in gastrocnemius from mice exposed to the pulse or continually induced. ± SEM; ***$p<0.001$, by one-way ANOVA.

C. Representative images of DUX4 (red), Laminin (green) and nuclei (DAPI, blue) immunofluorescent staining of TA muscle of iDUX4;HSA mice. Scale bar 100 µm. Note that rare DUX4 staining is detected only in the continually induced group.
